# Right atrial extension of inferior vena cava thrombosis after microwave ablation of colorectal liver metastasis: a case report

**DOI:** 10.3389/fonc.2026.1897189

**Published:** 2026-08-14

**Authors:** Yun Lin, Jiaoling He, Tingfang Zuo, Zhiwei Yu, Yewen Wang, Chen Zheng, Ruixiang Zhou, Yan Zhang, Jian Yang

**Affiliations:** 1 Fuzong Clinical Medical College of Fujian Medical University (900th Hospital of PLA Joint Logistic Support Force), Fuzhou, China; 2 The School of Basic Medical Sciences, Fujian Medical University, Fuzhou, China; 3 Fuzong Teaching Hospital of Fujian University of Traditional Chinese Medicine, Fuzhou, China

**Keywords:** case report, colon cancer, inferior vena cava thrombus, microwave ablation, right atrial thrombus

## Abstract

Thromboembolic complications after microwave ablation of colorectal liver metastases are rare but can be life-threatening. We report a 43-year-old woman with advanced colon adenocarcinoma and a synchronous right hepatic lobe metastasis who underwent laparoscopic right hemicolectomy combined with microwave ablation. Sixteen days post-procedure, imaging revealed an inferior vena cava thrombus extending into the right atrium. Despite therapeutic anticoagulation with low-molecular-weight heparin and warfarin, the right atrial thrombus paradoxically increased in size. Anticoagulation alone failed, and the patient successfully underwent thoracoscopic cardiac thrombectomy. Microwave ablation of liver metastases adjacent to the inferior vena cava or its major hepatic vein can trigger thrombosis with paradoxical upward extension to the right atrium, even during therapeutic anticoagulation. When anticoagulation fails, surgical thrombectomy may be lifesaving. This case underscores the need for high clinical vigilance for thromboembolic events after microwave ablation in high-risk anatomical settings and highlights the importance of a multidisciplinary management approach.

## Introduction

Microwave ablation (MWA) has emerged as a widely accepted, minimally invasive treatment option for colorectal liver metastases, particularly in patients with oligometastatic disease or those undergoing conversion therapy. Its advantages include low morbidity, short recovery time and the ability to preserve liver parenchyma. However, as with any interventional procedure, MWA is associated with a spectrum of complications, among which thromboembolic events are considered rare but potentially life-threatening (1, 2).

Herein, we report an unusual case of a 43-year-old woman with stage IVA colon adenocarcinoma and a synchronous liver metastasis who developed an inferior vena cava (IVC) thrombus shortly after combined laparoscopic right hemicolectomy and MWA of the hepatic lesion. The thrombus extended continuously from the IVC into the right atrium. Despite immediate and continuous therapeutic anticoagulation with low-molecular-weight heparin and warfarin, the right atrial thrombus paradoxically enlarged, rendering medical management ineffective. The patient ultimately required thoracoscopic cardiac thrombectomy. This rare sequence of events highlights a previously underappreciated risk of MWA. It serves as a cautionary note regarding the appropriateness of MWA in certain clinical contexts and underscores the need for heightened vigilance for thromboembolic complications even after seemingly uneventful minimally invasive procedures.

## Case presentation

**Figure 1:**
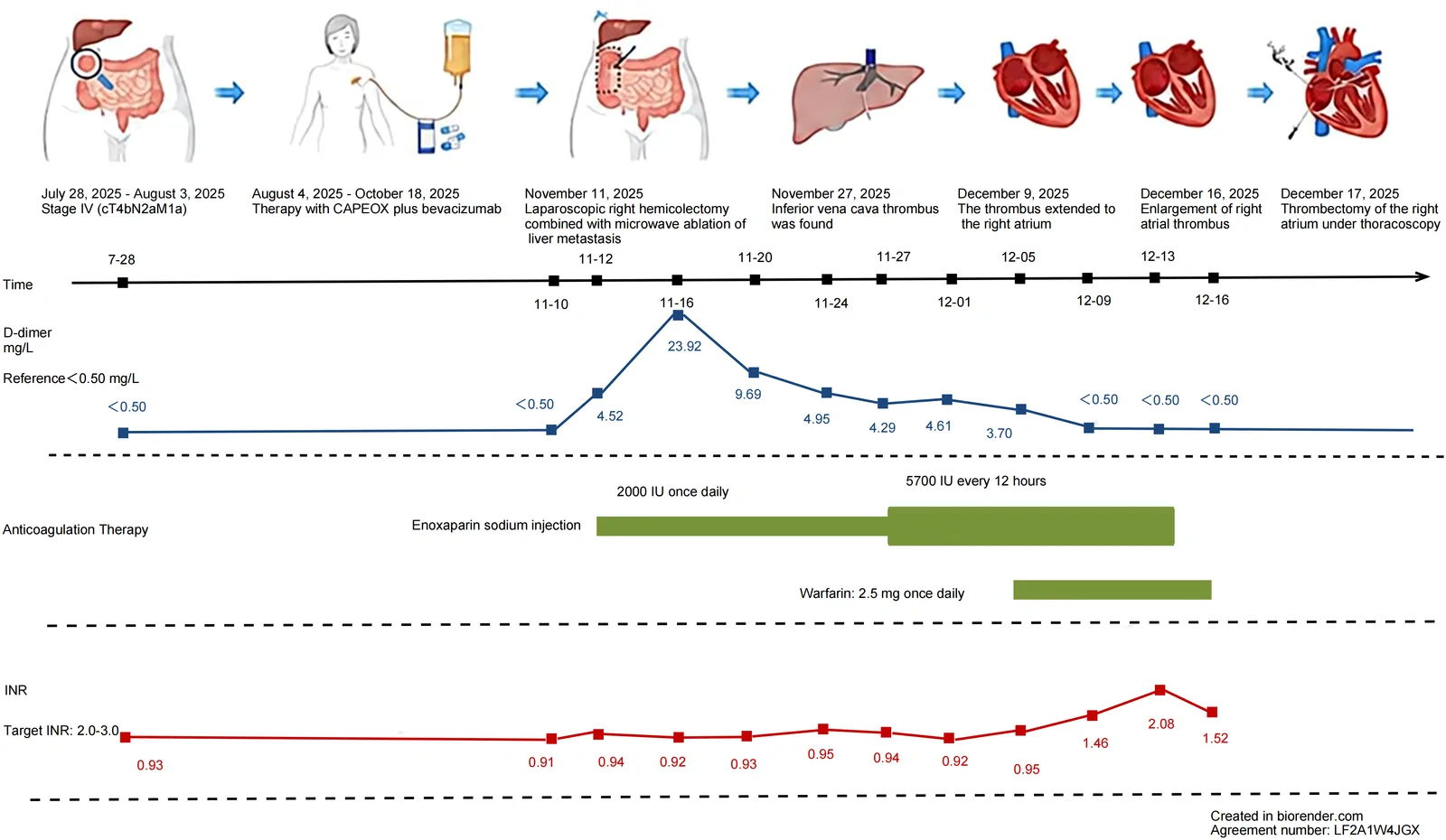
The timeline of diagnosis, treatment, and key laboratory findings.

A 43-year-old woman presented to our hospital on July 28, 2025, with abdominal pain and altered bowel habits with loose, pasty stools. The patient’s ECOG (Eastern Cooperative Oncology Group) performance status (PS) was 1. She had no significant prior medical or family history of cancer. Baseline laboratory testing showed tumor markers: carcinoembryonic antigen (CEA) at 2.01 ng/mL, carbohydrate antigen (CA) 12–5 at 8.65 U/mL and CA 19–9 at 23.70 U/mL. A colonoscopy subsequently revealed multiple colonic masses that exhibited an irregular surface with focal necrosis. The largest one was 13 × 10 mm. Histopathology confirmed invasive, moderately differentiated adenocarcinoma. Molecular testing indicated wild-type NRAS, KRAS, BRAF, and PIK3CA, proficient mismatch repair (pMMR). Abdominal computed tomography (CT) and magnetic resonance imaging (MRI) revealed thickening of the colonic wall at the hepatic flexure with luminal narrowing, accompanied by enlargement of five regional lymph nodes. The normal fat plane between the colonic wall and the perirenal space was lost, with obscuration of the renal capsular margin. In addition, a 31 × 25 mm mass was identified in the right hepatic lobe. According to the tumor, node, metastasis (TNM) staging system, the tumor was classified as stage IVA (cT4bN2aM1a) per the National Comprehensive Cancer Network (NCCN) guidelines (3). Regarding treatment, pMMR status is generally associated with a low response rate to immune checkpoint blockade. Hence, first-line therapy with capecitabine, oxaliplatin (CAPEOX) and bevacizumab started on August 4, 2025. **Figure 1** summarizes the timeline of diagnosis, treatment, and key laboratory findings.

Following six cycles of conversion therapy, the patient underwent laparoscopic right hemicolectomy combined with MWA of the hepatic metastasis on November 11, 2025. The liver metastasis was located in segments S7 and S8 near the hepatic dome, with a distance of 5 mm from the IVC. The ablation zone was positioned 1 mm from the IVC. Ablation was performed using a single antenna at 60 W for 5 minutes. The ablation zone completely covered the tumor lesion, with its left side closely abutting the IVC and its right side extending 5 mm beyond the tumor margin. The pathological stage after neoadjuvant therapy was ypT3N0. The procedure was uneventful. On postoperative day 1, the patient’s D-dimer level was elevated to 4.52 mg/L, and the Caprini risk assessment score for venous thromboembolism was > 5 (planned major surgery >45 minutes, malignancy and central venous access), placing the patient at high-risk for thrombosis (4, 5). Accordingly, prophylactic anticoagulation was initiated with enoxaparin sodium injection (molecular weight range 3500–5500 Daltons) 2000 IU administered subcutaneously once daily.

**Figure 2:**
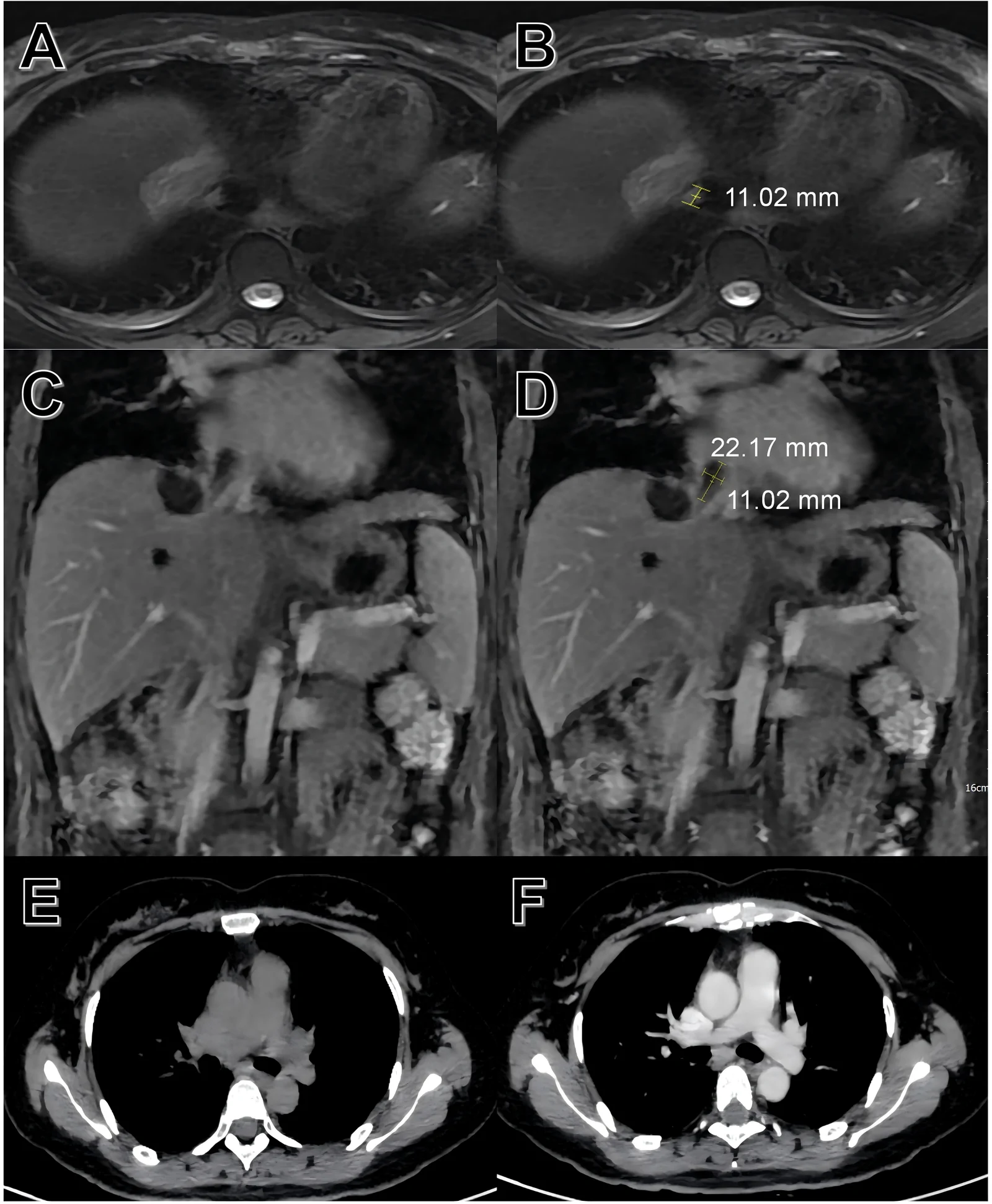
**(A–D)** abdominal magnetic resonance images obtained on November 27, 2025. **(A, B)** T1-weighted axial contrast-enhanced (T1-Ax-C+) images. **(C, D)** T1-weighted coronal contrast-enhanced (T1-Cor-C+) images. **(E, F)** CT pulmonary angiography images obtained on December 16, 2025.

**Figure 3:**
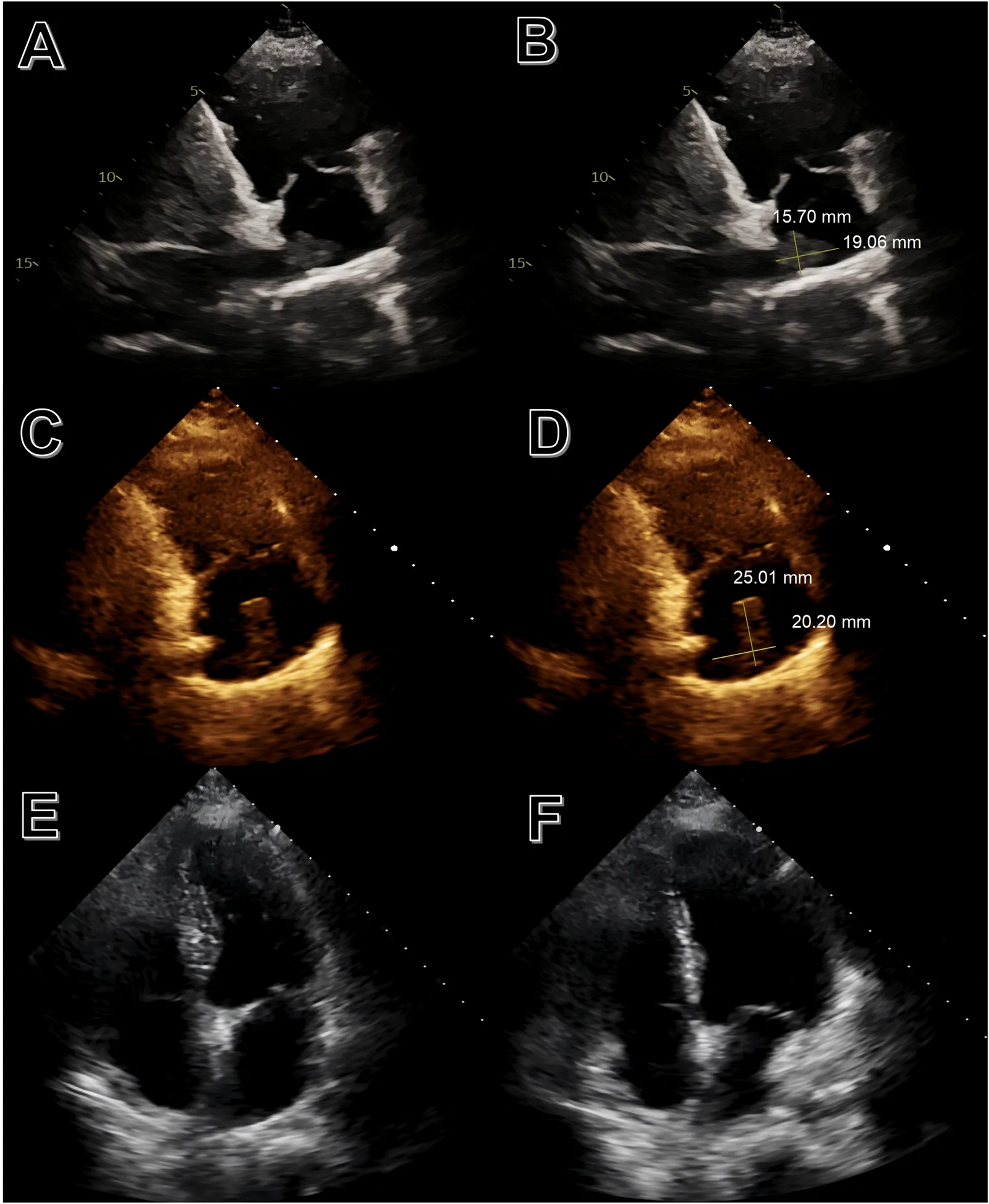
Serial echocardiographic findings. **(A, B)** echocardiographic images obtained on December 9, 2025. **(C, D)** contrast-enhanced echocardiographic images obtained on December 16, 2025. **(E, F)** echocardiographic images obtained on December 17, 2025.

**Figure 4:**
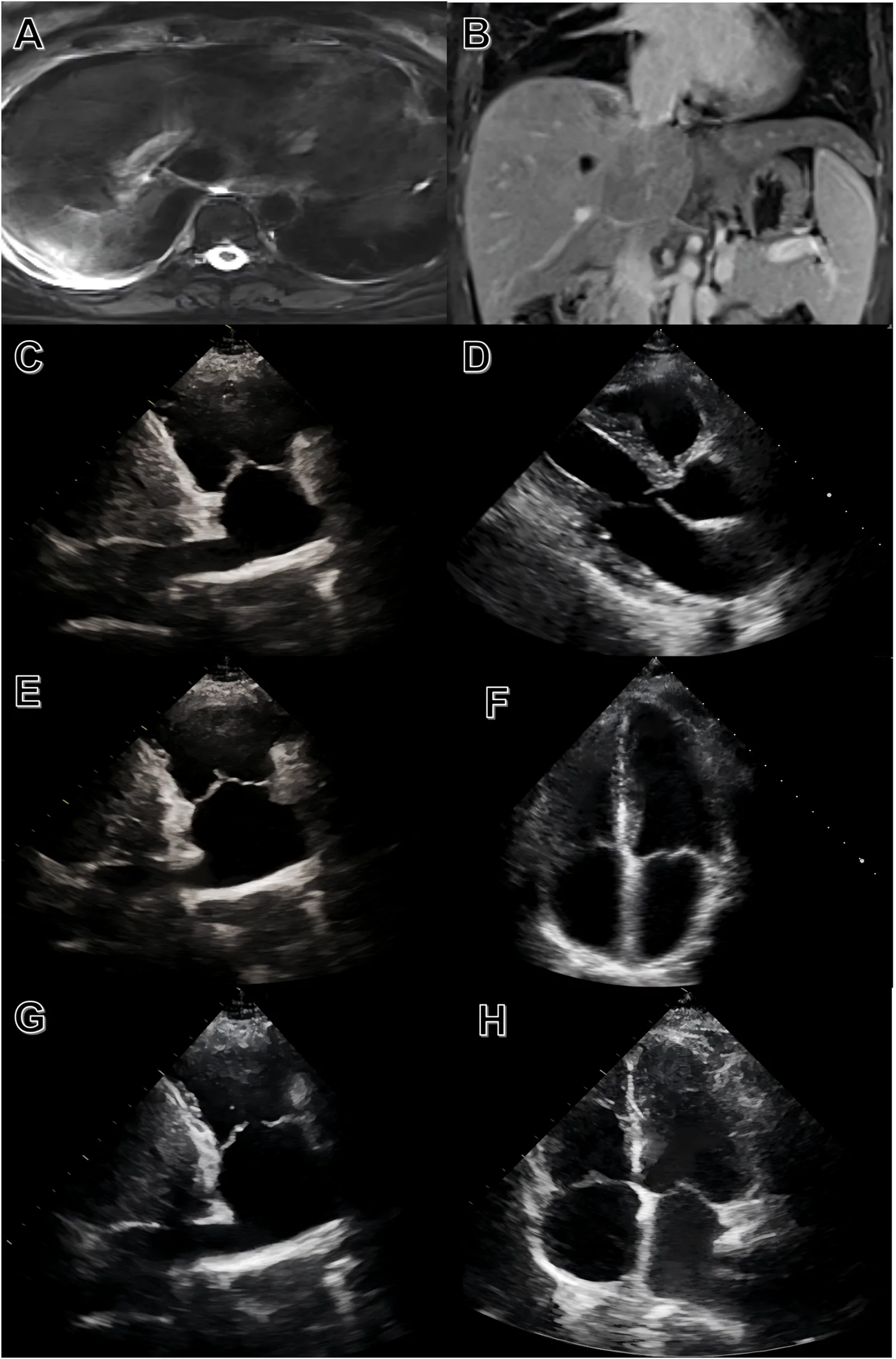
Postoperative follow-up images. **(A, B)** abdominal magnetic resonance images obtained on January 13, 2026. **(A)** T1-weighted axial contrast-enhanced (T1-Ax-C+) images. **(B)** T1-weighted coronal contrast-enhanced (T1-Cor-C+) images. **(C, D)** echocardiographic images obtained on February 3, 2026. **(E, F)** echocardiographic images obtained on March 17, 2026. **(G, H)** echocardiographic images obtained on April 28, 2026.

However, a routine MRI follow-up examination on November 27, 2025, unexpectedly revealed a thrombus measuring 22.17 × 11.02 mm within the IVC (**Figures 2A–D**). The D-dimer level was 4.29 mg/L. The patient was afebrile, with negative inflammatory markers and blood cultures. Liver and kidney function, along with platelet counts, have consistently remained within normal limits. Accordingly, enoxaparin sodium injection was adjusted to a therapeutic dose of 5700 IU (57kg, 100 IU/kg) every 12 hours. Subsequently, an echocardiogram performed following cardiology consultation revealed that the IVC thrombus extended into the right atrium, measuring 19.06 × 15.70 mm (**Figures 3A, B**). Given the possibility of an inadequate anticoagulant response to enoxaparin sodium injection, the regimen was transitioned to warfarin at an initial dose of 2.5 mg once daily, with subsequent dose adjustments based on the international normalized ratio (INR), and it was planned to discontinue enoxaparin sodium injection 24 hours after the INR reached the therapeutic range of 2.0–3.0 (22, 23). However, despite one week of continuous anticoagulant therapy, the right atrial thrombus paradoxically increased in size to 25.01 × 20.20 mm (**Figures 3C, D**) and exhibited increased mobility. The electrocardiogram consistently showed no significant abnormalities. CT pulmonary angiography (CTPA) was performed and ruled out pulmonary embolism (**Figures 2E, F**). Given the risk of pulmonary embolism from further dislodgement, a multidisciplinary consultation was convened. After weighing the risks of intraoperative bleeding against the imminent threat of thromboembolism, thoracoscopic thrombectomy was urgently scheduled within 24 hours (December 17, 2025). The operation was performed under general anesthesia. A right fourth intercostal space incision (1 cm) served as the main working port. A 4 cm longitudinal incision was made in the right groin to expose the femoral artery and vein, and cardiopulmonary bypass (CPB) was established via cannulation of the femoral artery and vein, along with the right internal jugular vein. After right atriotomy, exploration revealed a cord-shaped mass in the right atrium and at the orifice of the IVC, measuring overall 4.2 × 2.5 × 0.5 cm. The lesion was completely excised. Intraoperative echocardiography demonstrated no residual abnormal mass in the right atrium or at the orifice of the IVC (**Figures 3E, F**). Postoperative pathological examination confirmed the mass to be thrombotic tissue, which was reddish and loose, with scattered sparse epithelial-like cells in the periphery. Immunohistochemical staining showed: AE1/AE3 (–), CK5/6 (–), Calretinin (–), CK7 (–), CK20 (–), CDX-2 (–), SATB2 (–), CD163 (+), and IBA1 (+). The total operative time was 2 hours and 14 seconds. The patient was transferred to the intensive care unit postoperatively, and no significant surgical complications were observed. Warfarin 2.5 mg once daily was initiated 48 hours postoperatively, with subsequent dose adjustments based on the INR; the total hospital stay was 12 days. The follow-up abdominal MRI on January 13, 2026, showed a patent IVC (**Figures 4A, B**). Follow-up echocardiography on February 3, March 17, and April 28, 2026, showed no thrombus was detected in the IVC or right atrium (**Figures 4C–H**). The patient resumed palliative chemotherapy (CAPEOX and bevacizumab) in the next cycle and was still receiving it at the most recent follow-up.

## Patient perspective

The patient provided informed consent for publication. Postoperatively, she reported only mild fatigue but no chest pain, dyspnea or palpitations. Told about the incidental thrombus and the need for thoracoscopic thrombectomy, she initially felt anxious but was reassured by the multidisciplinary team. She resumed daily activities within one week after thrombectomy and has since remained on palliative chemotherapy without major complaints.

## Discussion

MWA is frequently used to treat hepatocellular carcinoma or metastatic liver tumors, favored for its ability to spare normal hepatic tissue and promote rapid convalescence. However, its expanding application underscores the need to recognize uncommon yet potentially life-threatening complications. Although rarely documented, venous thromboembolism following MWA is a clinically relevant concern. Currently, cases of hepatic vein and portal vein thrombosis following MWA have been reported (6–9). Additionally, Ma et al. (1) reported a case of colorectal cancer liver metastasis in which thrombosis occurred in the left hepatic vein extending into the IVC, with the thrombus tip reaching the right atrium two months after MWA. The thrombus resolved after two weeks of anticoagulant therapy. In contrast to their case, the patient in the present case report had a more advanced stage of disease, a larger liver metastatic lesion, and IVC thrombosis was detected as early as 16 days post-procedure, with rapid progression.

Several interacting mechanisms likely contributed to IVC thrombus development in our patient. Thermal endothelial injury may have played a local role, as the right hepatic lobe metastasis lay adjacent to the IVC. Thermal energy during MWA may denude the endothelium, expose tissue factor, and activate the extrinsic coagulation cascade. Meanwhile, this patient also harbored multiple strong prothrombotic factors that must be considered. Active metastatic colon adenocarcinoma (stage IVA) induces a hypercoagulable state via tumor-derived tissue factor (10), inflammatory cytokines (11), platelet activation (12), and impaired protein C pathway (13); the elevated D-dimer at detection reflected ongoing thrombin generation. Additionally, recent systemic therapy and prior bevacizumab exposure may have further compounded the thrombotic risk. The surgical context (laparoscopic right hemicolectomy 16 days earlier) contributed via inflammatory stress (14, 15), venous stasis (from pneumoperitoneum and immobilization) (16), and postoperative hypercoagulability. The unique anatomy, with hepatic veins directly draining into the IVC and the lesion adjacent to it, provided an anatomical conduit for thrombus propagation upstream into the cardiac chambers. Although the patient had an implantable venous access port, its tip was positioned in the superior vena cava, anatomically distant from the IVC thrombus; therefore, the likelihood of catheter-related factors is relatively low. Collectively, we interpret the event as likely multifactorial, with the MWA-related thermal injury representing a temporal association rather than a definitive causative factor.

Several differential diagnoses warrant consideration. The pathological examination of the resected mass confirmed it to be thrombotic tissue, with immunohistochemical staining negative for tumor-associated markers and positive for histiocytic markers, effectively ruling out a tumor thrombus or cardiac metastasis from the primary colon adenocarcinoma. The patient had an implantable venous access port in place for over three months with regular maintenance and no signs of port dysfunction, such as difficult aspiration or infusion. As such thrombi typically occur in the superior vena cava or the accessed vein (e.g., internal jugular or subclavian vein), whereas our patient’s thrombus was located in the IVC, anatomically distant from the catheter tip, catheter-related thrombosis was thus considered unlikely. The patient was afebrile, with negative inflammatory markers and blood cultures; the absence of valvular vegetations on echocardiography further argued against infective endocarditis. The thrombus exhibited an acute-to-subacute histological appearance, consistent with recent formation rather than a chronic organized thrombus. Overall, the diagnosis of a bland thrombus arising from the interplay of local thermal injury and systemic hypercoagulability remains the most plausible.

The most thought-provoking observation in this case is the extension of the IVC thrombus towards the right atrium, despite early anticoagulant interventions. Several mechanisms, acting in a temporal and interactive manner, may explain this therapeutic failure. First, cancer-associated hypercoagulability can affect the efficacy of antithrombotic therapy. Second, poor low molecular weight heparin response is suspected to stem from antithrombin deficiency or from nonspecific binding to proteins such as platelet factor 4, lipoproteins, factor VIII, and fibrinogen (17, 18). Third, warfarin initiation itself may have paradoxically favored thrombosis, as it depletes protein C, a natural anticoagulant, before reducing procoagulant factors, thereby creating a transient hypercoagulable milieu (19, 20). Fourth, thrombus maturation must be considered: as a clot ages, it transforms from a fibrin-rich acute structure into a chronic fibrotic mass that is poorly responsive to pharmacological lysis. Anticoagulants primarily prevent further accretion rather than dissolve established thrombus; thus, continued enlargement implies that new thrombus formation exceeds the inhibitory capacity of the regimen (21). Collectively, these systemic and local factors, rather than a single cause, best explain the relentless progression despite maximal medical therapy.

MWA is generally safe, but venous thrombosis may occur due to thermal injury to adjacent vascular structures or the thrombogenic effects of ablated tissue. To minimize this risk, meticulous technique is crucial. This case carries several additional practical messages. First, hepatic metastases adjacent to major hepatic veins or the IVC warrant heightened surveillance for venous thrombosis after MWA, including early contrast-enhanced imaging. Second, when thrombus is detected and extends toward the right atrium, prompt escalation beyond standard anticoagulation (considering thrombolytic therapy or surgical consultation) should be pursued, as anticoagulation alone may fail. Third, multidisciplinary management (interventional radiology, hematology, cardiothoracic surgery and oncology) is essential to balance thrombosis progression, bleeding risk, and oncologic priorities.

The case has several limitations. First, definitive causality could not be established. Second, neither protein C, factor VIII, nor anti-Xa activity was monitored to determine the cause of thrombosis or to assess the efficacy and safety of anticoagulant therapy. The lack of serial imaging immediately after MWA precludes precise determination of thrombus onset timing, and the single-case design inherently limits the generalizability of our observations.

## Conclusions

This case suggests that MWA adjacent to the IVC may predispose to thrombosis with right-atrial extension, and that therapeutic anticoagulation may fail to halt its progression. The prothrombotic state, potentially driven by thermal endothelial injury, postoperative inflammation, venous stasis, and cancer-associated hypercoagulability, together with the direct venous drainage anatomy, may have favored upstream clot propagation. Although proof is lacking from a single observation, these factors collectively generate the hypothesis that early imaging as well as surgical readiness are critical in such settings, and multidisciplinary care is advised.

## Data Availability

The original contributions presented in the study are included in the article/supplementary material. Further inquiries can be directed to the corresponding authors.
